# Winter Retreat for the Consumptive

**Published:** 1840

**Authors:** 


### WINTER RETREAT FOR CONSUMPTIVES.

Some time since we received a letter from Dr. John L. Sullivan, of New Haven, Ct. accompanying a public printed communication to the physicians of New England on the peculiar fitness of the *south* side of the Island of Cuba, as a winter residence for the consumptive. The small city of *Trinidad* is most eligible.

"The peculiarities of this locality justify this preference. The town is situated on the south side of a range of mountains from east to west, on elevated ground four miles from the sea. It is built of stone, and is paved, and it is watered by a clear and *rapid* river that flows near. Thus sheltered from the north winds by these high mountains, that intercept and attract the moisture of the sea air, it enjoys a peculiarly dry elastic atmosphere, of an equable and mild temperature. As a proof of it, exercise can be borne by invalids without causing debility.

This place can be reached by a voyage direct; vessels are often going out; or, by the way of the Havana, from whence there is already a rail-road, thirty miles out of forty-two, towards the town of Batabano; and thence a steamboat runs within islands to *Casilda,* its sea-port. Besides that, British steam ships of the larger class, are soon to run between New York and the Havana.

This facility of communication permits us to consider Cuba as accessible by sea as the southern ports, and from New Orleans and Pensacola very accessible from the Western States."

Convinced as we are, that the States of Louisiana, Alabama and Georgia, offer but illusory retreats for the consumptive, for a great part of the winter being too cool and damp, we must, until tropical Florida can be prepared and openened to our patients, advise them to go beyond the United States. In doing so, it is, obviously, better to choose the south than the north side of that island to which most of them necessarily go, and too often return in no degree benefitted. D.

